# Navigating towards strengthened climate service processes

**DOI:** 10.1007/s13280-025-02136-6

**Published:** 2025-02-08

**Authors:** Lotten Wiréhn, Gustav Strandberg

**Affiliations:** 1 Department of Thematic Studies – Environmental Change, Linköping University, 581 83 Linköping, Sweden; 2 Centre for Climate Science and Policy Research, Linköping University, 581 83 Linköping, Sweden; 3 Rossby Centre, Swedish Meteorological and Hydrological Institute, 601 76 Norrköping, Sweden

**Keywords:** Climate change, Climate projections, Co-creation, National climate service, Recommendations, Science communication

## Abstract

Despite the importance of salient, credible, and legitimate climate information for climate action, studies demonstrate a persistent usability gap between the information provided and what users find relevant and useful. Drawing from scientific literature and our experiences working with a Swedish national climate service, we explore and reflect on challenges and barriers with climate services using an analytical framework of four pillars. Based on this, we provide four overarching recommendations (and fundamental needs): (i) Advancing data production and analysis, (ii) Establishing a climate service collaboration forum, (iii) Fostering active users, and (iv) Prioritising long-lasting funding. These recommendations are directed to policymakers and the climate service community to transition the production and use of climate information from short-term studies and initiatives to long-lasting processes. We argue that adopting these recommendations can support climate-resilient development through strengthening climate service infrastructure and enhancing capabilities and skills of the actors involved.

## Introduction

The requisite for climate information to support decision-making has been emphasised since the beginning of the twenty-first century, both within and outside academia. Climate information refers to information about the “past, current, or future state of the climate system that is relevant for mitigation, adaptation, and risk management” (IPCC 2021 p. 172). Estimates of possible future climates, i.e. climate projections, are especially important to understanding and assessing climate-related risks and adaptation needs to climate variability and change (Hewitt et al. 2021).

In 2009, the Global Framework for Climate Services was established by the World Meteorological Organization to promote the development of information and services for societies to better handle climate risks and opportunities (WMO 2014). Since then, research and development on climate services^1^ have escalated, including studies on realised services and climate service characteristics, alongside criticism that the successfulness or effectiveness rarely are evaluated (e.g. Vaughan and Dessai 2014; Boon et al. 2022). It has also been declared that climate services are crucial to assist decision-making processes at all levels of society towards climate-resilient development (e.g. Hewitt et al. 2021). Nevertheless, while effective climate action could be argued to rest on credible climate data and information, this information must be relevant and usable for stakeholders and decision-makers to support action (Kirchhoff et al. 2013; Jebeile 2024). This is where great challenges have been demonstrated for more than a decade, commonly recognised as the “usability gap” (Lemos et al. 2012). To this point, climate service scholars repeatedly come to a similar conclusion that the provision and use of climate information involve difficulties that hinder applicability and usability, and deem an increased need for co-creation and stakeholder engagement (e.g. Hansen et al. 2019; Jacobs and Street 2020; Malakar et al. 2024). However, despite the common acknowledgement of adopting co-creation in climate service processes, issues regarding the usability gap seem to remain.

In this paper, we ask why this is and argue that if the notion that climate action is underpinned by salient, credible, and legitimate climate information (e.g. IPCC 2022 TS.E; Malakar et al. 2024) is to be maintained, society must start prioritising climate services at multiple levels. If trust in the importance of climate services for well-informed decision-making does not hold, the concept of climate services could be reconsidered. However, given the current knowledge, addressing challenges and barriers to improve climate services and close the usability gap remains essential.

Drawing upon scientific literature, insights from stakeholder dialogues, and experiences working with climate service processes in Sweden (e.g. Strandberg et al. 2024; Wiréhn 2024), this paper examines and reflects upon climate service challenges and barriers, with focus on climate information of multi-decadal regional climate projections.^2^ With this as a foundation, we present recommendations that aim to strengthen climate service processes at national levels to foster more effective and viable development and use.

### The Swedish climate change scenario service

**Fig. 1:**
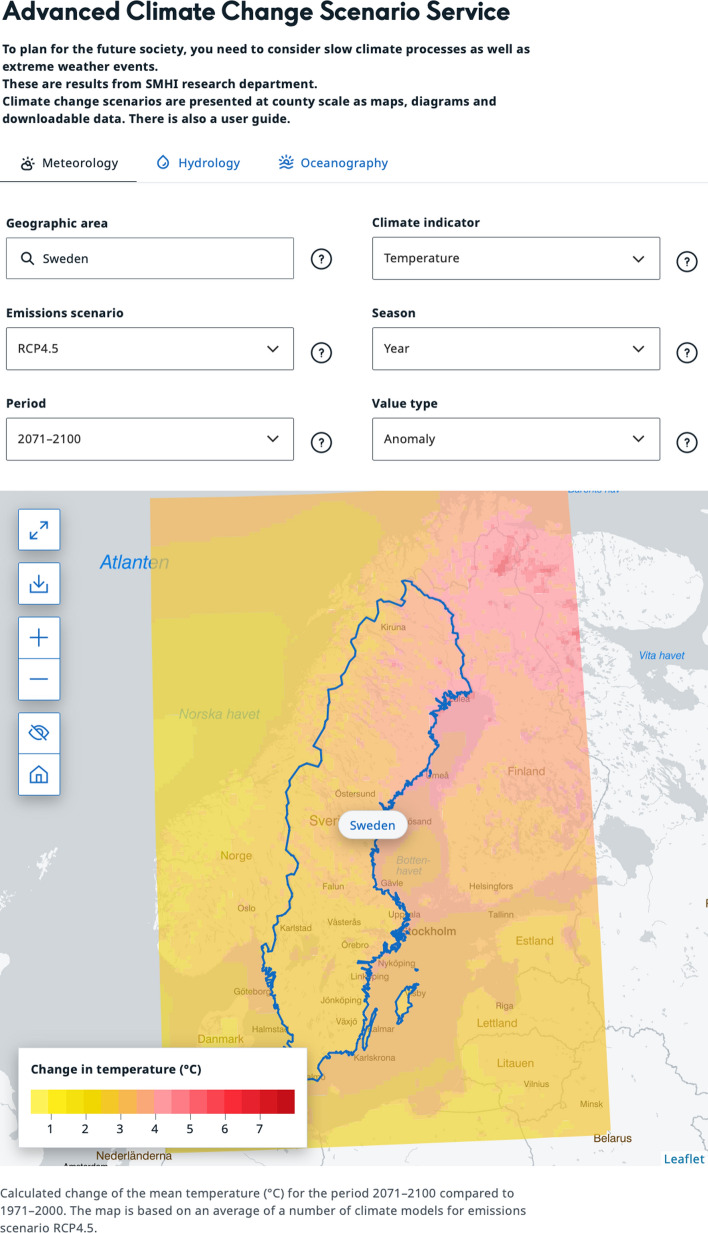
Screenshot from the website of KST - the Swedish climate change scenario service (from July 2024) (SMHI 2024)

The Swedish Meteorological and Hydrological Institute (SMHI) is the provider of the Swedish Climate Change Scenario Service—KST^3^ (SMHI 2024). The service was launched in 2021 and stems from the demands from Swedish stakeholders and the need to provide updated climate change projections for use by society. The climate service approach including the provided climate change projections is an ongoing continuation of the work of Kjellström et al. (2016). The latest generation of the service includes different types of climate change information in a uniform approach through maps, graphs, text, and tabular data, separated under three tabs of meteorology, hydrology, and oceanography, Fig. 1.

The reflections connected to the KST in this paper refer to the tab with meteorological variables in the service. That includes estimates of possible future climates until the end of the twenty-first century, i.e. simulated climate change projections, relative to historical observations for the period 1961–2018. As the national climate service provider and the dedicated national expert agency for weather, climate, hydrology, and oceanography of Sweden, SMHI has long experience in climate modelling and communication with a wide range of different user groups. Although Sweden as a country, and SMHI as a national expert agency, could be considered to be at the forefront of regional climate model projections and climate services (Kjellström et al. 2016), especially in contrast with developing countries’ capacity (Mahon et al. 2019), a range of barriers and challenges for climate service processes also exist for Sweden (Donnelly et al. 2018; Ernst et al. 2019). The authors’ experiences and lessons learned from working with the development and use of the KST are in this paper aligned with challenges and barriers as identified in the international scientific literature on climate services.

### Four pillars of climate services

**Fig. 2:**
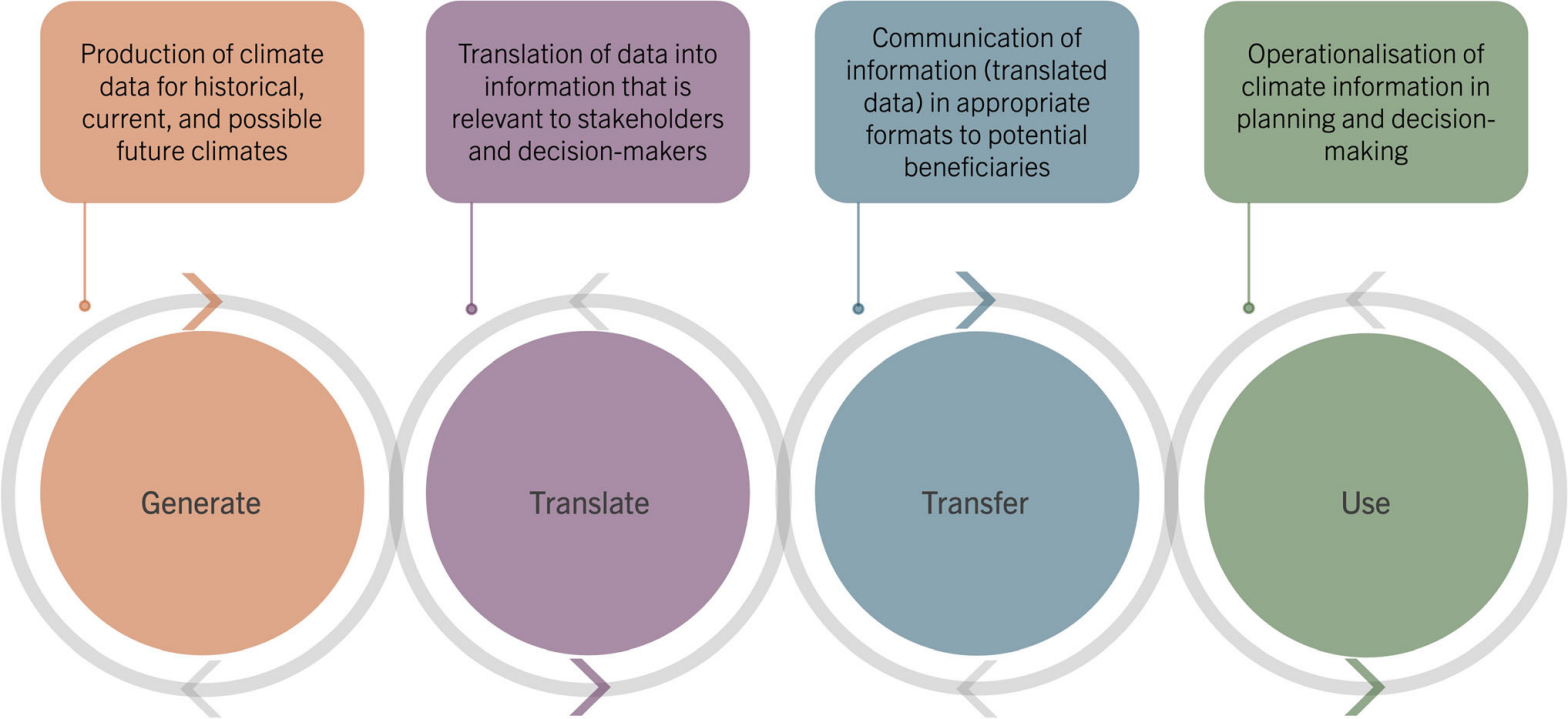
Conceptualisation of the four pillars of climate services in an iterative co-creation processes, adapted from Grossi and Dinku (2022) and IRI (2024)

The scientific literature commonly agrees that climate services are not solely products supplied to end-users; rather, they should include an iterative co-creation process among climate information producers, users, and other stakeholders, working towards bridging the frequently recognised usability gap (Lemos et al. 2012) and ensuring the provision and use of relevant, valid, credible, equitable, and accessible climate information (e.g. Vincent et al. 2018; Máñez Costa et al. 2021). In this paper, we interpret the concept of “co-creation” of knowledge as the integration between science and society, encompassing the phases of co-design, co-production, co-dissemination, and co-evaluation of climate information and services, where ideally, reflexive learning should occur continuously among the involved actors (Mauser et al. 2013; Bremer et al. 2019). One way to understand climate services beyond the mere production of climate information is through the four pillars: *generate*, *translate*, *transfer,* and *use* (Grossi and Dinku 2022). This four pillars framework is a means to highlight the important iterative and perpetual co-creation processes of climate services while not underplaying the value of generating high-quality climate data (ibid). This paper interprets *generate* as the production of credible and legitimate climate data; *translate* as the translation of data into climate information (e.g. indicators, aggregations) that are relevant and useful to stakeholders and decision-makers; *transfer* as the communication and accessibility of information in appropriate usable formats to potential beneficiaries (users); and *use* as making the climate information usable and operational in planning and decision-making (Fig. 2). Preferably, different types of co-creation should be incorporated into all of these pillars, where appropriate, from the design of the “problem” and data to be generated to the production of services that integrates the climate information with other contextual information to support planning and decision-making (e.g. Máñez Costa et al. 2021).

The pillars of translate and transfer are closely related in terms of developing user-relevant climate services, but the transfer pillar could specifically be seen as how data and information are made practically usable based on how it is communicated (cf. Dilling and Lemos 2011). The aspect of accessibility is important in this regard, including the users’ possibility to obtain information and how information is communicated in terms of language, visual representations, and data formats (Dilling and Lemos 2011). All four pillars are indeed interlinked and dependent on each other but in this paper, we employ them as discrete analytical lenses when scrutinising climate service challenges and barriers. Drawing on scientific literature and our gained insights from working with the KST, we outline key challenges and barriers relative to the four pillars.

## Unresolved challenges and barriers

Aligned with the four pillars of climate services, this section outlines and discusses ten themes of challenges and barriers that in different ways can explain the persistent usability gap, and thus merit further attention and action.

### Generate

#### Model skill and uncertainty

A key climate change information challenge is the uncertainty in the model projections that stems from factors such as the societal development (the emission scenarios), climate system variability, and model responses to anthropogenic forcing (Hawkins and Sutton 2009; Brunner et al. 2019). Since projections are based on models using different physics, parameterisations, etc., there is a spread in the model outputs even if the simulations are based on the same emissions scenario (Donnelly et al. 2018). This spread is often presented as the uncertainty, but that can be criticised because it does not represent the “real” uncertainty (Donnelly et al. 2018) as in the variance of prediction errors (Hawkins and Sutton 2009). Since the relative frequencies of simulations do not reflect the probability, it has been argued that such estimates do not provide information about the reliability of climate change projections (Donnelly et al. 2018; Nissan et al. 2019).

In the KST, data are built on the EURO-CORDEX Regional Climate Model (RCM) ensemble at 12.5 km horizontal resolution (Jacob et al. 2020), which currently is the best available climate scenario data for Europe. However, using non-adjusted EURO-CORDEX data for national level climate indicators is inadequate (Jacob et al. 2020; Vautard et al. 2021), especially when climate indicators are defined using absolute thresholds, as in the KST case. Therefore, bias adjustments are implemented to address this (Berg et al. 2022). Despite the high-quality data used for the KST, the challenge of model skill and uncertainty is still acknowledged. The decision has been made to use the full EURO-CORDEX ensemble as this large RCM ensemble can better reflect the spread of results. However, such an ensemble is also called an “ensemble of opportunity” (Tebaldi and Knutti 2007), meaning that the full ensemble is used without considering its balance or the possible overrepresentation of certain models or model combinations, nor is the quality of individual ensemble members considered. With an unbalanced ensemble, there is a risk of not capturing the climate sensitivity correctly. Nevertheless, using larger ensembles, such as CMIP, provides a better statistical basis for exploring and explaining model “uncertainty” in more detail.

From the usage perspective, the level of uncertainty, reliability, and credibility of model projections is key (Hawkins and Sutton 2009; Jebeile 2024). We assert that these qualities affect the entire climate services process and must therefore be ensured to make services usable in decision-making processes. However, even when the best available climate projections and bias adjustments are ensured, interpreting the spread and uncertainty of the projections risks perpetuating the usability gap (returning to that under *transfer*). Maintaining high model skill and minimising uncertainty is also very time-consuming. It involves developing methods, performing calculations, massive data processing, quality control, and handling various technical issues due to different metadata and formats of the processed data files. The time and capacity for this work need to be better accounted for in the climate service development. Thus, basic climate modelling research needs ongoing funding to ensure the credibility and legitimacy of the climate modelling data. Although it probably varies between countries and institutions, there is a tendency for such climate science funding to be a limitation today, which risks undermining the co-creation efforts to close the usability gap.

#### Resolution and downscaling

Literature shows that disparities often exist between the available data with adequate skill and quality, and the spatial and temporal scales required by users of the information. If the available data does not meet the specific needs of users, it adds to the usability gap. For example, while the skill of climate simulations often is better at larger spatial scales, users—such as decision-makers—strongly regard it more useful with climate projections at smaller scale, e.g. sub-city, (Dilling and Lemos 2011; Ernst et al. 2019; Jacobs and Street 2020). An important related challenge is that high-resolution simulations require significant computational resources (e.g. Ernst et al. 2019). The demand for such data must be balanced against the high computational cost and data volume (e.g. Smid and Costa 2018). This connects back to the uncertainty challenge, as it is not feasible to have large model ensembles of downscaled high-resolution simulations. Therefore, national climate service providers must consider the varying values of different types of climate projection data while engaging in dialogues with users to make prioritisation regarding geographical and temporal focuses. However, the multitude of methodological decisions, such as determining the number and selection of GCMs and RCMs to include in climate simulation ensembles, choosing downscaling methods and models, and applying bias adjustment approaches (e.g. Berg et al. 2022; Tanimu et al. 2024) is challenging for climate data providers due to the lack of state-of the-art guidelines (e.g. Donnelly et al. 2018).

The KST has not experienced significant downscaling and high-resolution challenges so far, as it has focused on bias-adjusted EURO-CORDEX RCM ensembles. However, given user demands, downscaling techniques, and computational advancement, we assert that climate service providers must make advancements in approaches to combine RCM simulations with downscaled higher-resolution climate data to increase the usability while making the best out of the available data and information.

### Translate

#### Absent mutual understanding

There is a common call for climate information providers to better understand user contexts (e.g. Dilling and Lemos 2011; Jacobs and Street 2020). Jacobs and Street (2020) for example argue that climate scientists often assume users’ needs and presume that the produced information will be utilised. Such assumptions, even if not always the case, are problematic as they likely contribute to the persistent lack of common understandings, and thus, the usability gap.

Through KST, SMHI works to foster understanding in both directions between providers and users of climate projections by establishing ongoing collaborations with governmental agencies. However, these collaborations have often been ad hoc or based on personal relationships. We mean that to create the integration of science and society for climate services—crucial for mutual learning, understanding, and ultimately usability—these collaborations should be institutionally organised. Without institutionalised collaborations, it is difficult to work consistently with co-creation to address challenges and barriers explaining the usability gap.

The lack of mutual understanding remains a challenge also in the KST case. On the one hand, providers, typically climate scientists, possess profound knowledge of the climate system and have technical expertise to handle extensive data and related uncertainties. Users, such as, stakeholders from county administrative boards or extension officers, on the other hand, have critical sectoral expertise that climate scientists are not familiar with, but which is important for translating data into relevant and usable climate information (Wiréhn 2024). Users typically spend only part time on climate or climate adaptation in their work, if any dedicated time at all. Naturally, they have a lesser understanding of climate data and lower technical and interpretative abilities compared to climate scientists. Nevertheless, while providers must learn about how data can be interpreted, used, and incorporated into decision-making contexts, users need to acquaint themselves with how to interpret data and be aware of its limitations, which currently tend to be a barrier (Wiréhn 2024). Thus, we urge for improved capacity building among users to comprehend how the information can be used and its limitations in their planning and decision-making (e.g. Dilling and Lemos 2011; McNie 2013), which could be part of the institutionally organised co-creation for mutual understanding.

#### Defining users

Users and other stakeholders must be identified and engaged already at the initial stage of co-creation to involve them in the design, production and evaluation of services to ensure relevance, trust in the service, and to evaluate the actual usability of the service (e.g. Lemos et al. 2012; Baulenas et al. 2023; Kalsnes et al. 2023). However, climate service user groups are not homogeneous; even within organisationally defined groups, there are often diversities regarding objectives and priorities (Buontempo et al. 2018). This makes user definition and selection a significant challenge that requires a systematic approach (Baulenas et al. 2023).

In the KST, there is a phrasing indicating that the intended user is anyone who works with questions concerning planning for our future society: “To plan for the future society, you need to consider slow climate processes as well as extreme weather events” (SMHI 2024). Given the need to define users and increase their usability through co-creation involvement, this presents a particular challenge for *national* climate services, as these are typically directed to a very wide range of potential users with different needs (i.e. Kundzewicz et al. 2017). SMHI addresses this challenge by working in close dialogue with a prioritised user group, the Swedish County Administrative Boards and other governmental agencies. Specifically, the objective is to gain insight about climatic indicators related to relevant sectoral impacts and adaptation needs. As these boards administer the climate adaptation work in Sweden, focusing on them is justifiable instead of attempting to address the broad range of potential users and all their needs. We believe that identifying such prioritised user groups and systematically collaborating with representatives is essential to improving co-creation in national climate services.

#### Level of indicator tailoring

Finding the most appropriate level of indicator tailoring involves balancing the needs of specified users with the ability to mainstream a service, in co-creation with the intended users (e.g. Buontempo et al. 2018). Indicators need to be defined so that they are neither too general nor too focused on a single factor (Ernst et al. 2019). This balance is especially challenging and important for national climate services, given their wide range of potential users and the different relevant and usable contexts (Jacobs and Street 2020).

This challenge has been experienced in the KST case. The often quite specific indicators and analyses requested by users are problematic to include given the general focus of KST. Furthermore, providing a comprehensive set of all relevant climate indicators for every potential user is likely unfeasible (i.e. Donnelly et al. 2018), and would make the service difficult to use. Thus, the challenge of balancing the generality of the national climate service with the often detailed “wish lists” provided by users could hinder its usability, thereby requiring new and structured approaches (i.e. Kundzewicz et al. 2017). Moreover, in the KST case, there are certain limitations to what the service can provide in terms of integrating climate data with sectoral contextual data. As the national climate service provider, it is currently not feasible for SMHI to present data of climate change impacts and associated adaptation needs for particular sectors. Such service would require moving beyond co-creation of climate data and information into information that incorporates data and perspectives from multiple research fields, sectoral experts, and various governmental authorities—all within a single service. In practice, coordination and organisation for such integrated climate services at national level is likely not realistic. The contextualisation in climate services is nevertheless key and must be addressed to the extent possible. We think that a realistic approach of contextualising climate information in national climate services today is through novel approaches of *how* the climate data and information is represented (*translate*) and communicated (*transfer*) (Guido et al. 2020; Wiréhn 2024).

#### Level of data precision and aggregation

A key challenge in the translation of climate projection data involves managing the data constraints and presenting data at the most appropriate level of precision (temporal and spatial aggregation). The mismatch between the precision of the provided data and user demands limits its perceived relevance (e.g. Donnelly et al. 2018; Wiréhn 2024). One example concerns the temporal aggregation of model outputs into a more user-relevant indicator, like weeks, instead of season, which, however, can be problematic for RCM ensembles (e.g. Nissan et al. 2019).

It is critical that representations of climate change projections do not create an illusion of precision that is not valid, which could result in decision-makers being overconfident (Nissan et al. 2019). In KST, non-bias-adjusted variables are not included in any climate indicators because they would not be reliable. Preferably, when a balance between user needs and model limitations have been identified, the co-produced climate information should be evaluated to assess its benefits to support climate adaptation, but this is currently lacking in climate service processes (e.g. Suckall and Soares 2022). Similarly, we see a need in the KST case to have more communication of what the existing data resolution can offer. Higher resolutions add details but generally do not alter the key messages. While users often request higher resolution, the existing resolution would many times be sufficient to answer users’ questions concerning, for instance, trends.

KST intends to work with the challenges of requested tailoring of climate information by viewing the service as a process and not merely data provision to users. If climate data are reviewed by users and other stakeholders with sector expertise—preferably together with climate experts—they learn from each other, as one part know about the possibilities and limitations of climate model data, and the other part know about challenges and potential consequences for the respective sectors (Strandberg et al. 2024). Such mutual learning dialogues have existed in the case of KST, but there is currently no structured coordination and assigned responsibilities for this, which tend to make the meetings and dialogues somewhat ad hoc and not as persistent as desired.

### Transfer

#### Interpretation support

Given the uncertainties and limitations of climate models, it is challenging for providers to represent climate information and for users to interpret it (Ernst et al. 2019). Since climate scientists are familiar with the data, it is reasonable to suggest that they should have the responsibility for providing interpretation support (Donnelly et al. 2018). However, despite knowing data limitations and uncertainties, making this support user-friendly is very difficult (Donnelly et al. 2018; Ernst et al. 2019).

In KST, the ambition is to incorporate clear and meaningful communication of uncertainty and model spread, but it remains a significant challenge to determining the appropriate level of detail about model limitations and uncertainties, likely true for many national climate services given their differences (i.e. DMI 2024; DWD 2024; NCCIS 2024). While evaluating ensemble statistics and other complex analyses—such as the representativeness of the ensemble compared to the larger CORDEX and CMIP ensembles or the variability in large GCM ensembles—is necessary for maintaining scientific quality and supporting appropriate interpretations, our experience is that users often do not appreciate such complex data. Instead, a multifaceted presentation of ensemble statistics could potentially discourage usage altogether. Nevertheless, users must accurately interpret the climate projections.

In KST, the decision has been made to present climate indicators first, while information explaining RCP scenarios and uncertainties through text, error bars, ensemble spread, and standard deviation is provided as complementary information. Again, co-creation is essential for understanding how users perceive the climate projections and uncertainties, where the collaborations can inform communication approaches to enhance the usability and ensure informed decisions. We see a need for continued and advanced research as well as societal attention on approaches to making the complexities of climate data more accessible and understandable to users.

#### Service standards and maintenance

Despite the mounting development of climate services (Boon et al. 2022), there are currently deficient standards and frameworks for climate services (Hewitt et al. 2021). It is crucial for national climate services on climate projections to use common terminology for climate data, supporting information, and data formats (ibid). Without widely acknowledged standards, there is a risk of services misrepresenting climate information, which can lead to user misunderstandings and misuse (Filho 2020; Hewitt et al. 2021).

Regarding data format, converting data from climate model formats (usually NetCDF) to more accessible geographical information formats (usually shapefile/raster file) take time and increases the data volume. Moreover, GIS formats do not always work well with gridded data, large data volumes, or four-dimensional data. A full-fledged data portal, allowing users to download “any” climate data in “any” format would require significant maintenance and larger computer resources. This issue links back to the crucial matter of defining users and their purposes. Some European countries do provide NetCDF climate projection data (DMI 2024; DWD 2024), likely directing the service to climate impact researchers rather than authorities for adaptation support. National climate service providers would benefit from clearer guidance on making climate service development decisions (in co-creation) and, depending on the choices made, have specific standards that can serve as quality assurance to avoid misrepresentation of data and misuse.

Significant challenges are also related to the constant need for climate service maintenance. A continuous inflow of data increases the requirement for service maintenance and adds pressure on how to *translate* and communicate the latest simulated climate data (Dilling and Lemos 2011). This new data can either complement or replace former ones. Up-to-date services should be the intension for all national climate service providers, but dynamic services require more maintenance than static ones. Each update must also be communicated clearly, explaining the motivation for the change and differences compared to the former data. The challenge often lies in the funding and allocating time for maintenance. For example, SMHI receives some state funding for KST, but these are annual grants. This makes long-term planning and strategy development difficult, limiting the possibilities to plan for advancement towards a more usable service.

### Use

It becomes especially evident that the four pillars coincide when considering the use pillar, as the efforts and processes connected to the other pillars affect the usability potential. However, there are some challenges and barriers that we specifically associate with the use pillar.

#### Institutional barriers in user organisations

We call for increased integration of climate information into decision-making processes that not necessarily concerns climate issues specifically but nevertheless depend on or effect climate. However, in decision-making for policies, strategies, assessments, and plans, climate information competes with many other types of decision-support information that may be more closely connected to the goal at hand (Dilling and Lemos 2011). For example, some agricultural extension officers in workshop dialogues where the KST was applied noted that farming, and thus extension, depends on many factors other than climate, such as soil characteristics and policies, making these factors more important to account for in planning than climate projections (Wiréhn 2024). Moreover, political discourses likely play a role. If government agencies prioritise targets other than those related to climate, climate information will probably be implemented to a lesser extent in decision-making.

Informal and formal institutional rules and traditions is a known climate information usage barrier (Dilling and Lemos 2011). It can include a lack of time, willingness, and the organisational culture regarding how to perceive and handle data uncertainty in decision-making (Dilling and Lemos 2011; Wiréhn 2024). While data uncertainty limits usability (Jacobs and Street 2020), it is not necessarily the uncertainty itself that is always the barrier, but rather the user’s capacity to interpret and understand the data (e.g. Donnelly et al. 2018; Wiréhn 2024). Thus, providing stakeholder capacity building efforts is essential to build trust in the usefulness of the information and stakeholder organisations’ capacity to take in, understand and use the information (e.g. McNie 2013). Users’ lack of time not only hinders actual use, but also the entire co-creation process. This needs to be addressed at both funding level and the user organisational level if the usability gap is to be bridged.

While many barriers could be addressed in the generate, translate, and transfer pillars, challenges concerning competition between different types of decision-support information, willingness, and lack of time are more difficult to address within these processes. If usability challenges, such as improved contextualisation of the information, providing interpretation support, and capacity building, were effectively addressed, institutional rules and traditions might not hinder usage. However, evaluating the causality of usability factors is difficult, and we are not aware of any such study. Nevertheless, acknowledging institutional barriers allows us to approach the usability challenge from different perspectives. As willingness and lack of time are challenges related to users’ organisations (i.e. Dilling and Lemos 2011), it is crucial to incorporate the use per se in the climate service process (Vaughan and Dessai 2014; Boon et al. 2022). However, this should not solely be ad hoc co-creation with selected representatives; authorities and organisations must be completely on board and integrate the climate information use into their management processes when relevant.

#### Trust and rigorous user engagement

Despite the emphasised importance of co-creation for increased usability within the climate service community (e.g. Máñez Costa et al. 2021; Strandberg et al. 2024), the processes themselves present considerable challenges. In this regard, a lack of trust is a key challenge that can hinder engagement.

“Trust” encompasses users’ perceptions of information legitimacy (Dilling and Lemos 2011) and trust in relationships between users and providers concerning knowledge exchange and data sharing (Ernst et al. 2019; Findlater et al. 2021). Although the climate service community generally favours bottom-up and demand-driven approaches, few climate service organisations attempt or have the capacity for rigorous user engagement (Findlater et al. 2021). Identifying stakeholders who could potentially benefit from the climate service and engaging them in development activities and use of climate information is often challenging (Dilling and Lemos 2011; McNie 2013; Ernst et al. 2019). Demonstrating the integrated value of co-creation of climate services more effectively to users during the early phases is likely one approach to enhance their interest in continued engagement.

User engagement is important in the KST case, but the responsibility and coordination of this engagement have not been formalised. It has relied on existing collaborations with governmental agencies, specifically related to climate adaptation. Nevertheless, SMHI’s continuous contact with the County Administrative Boards has been fruitful in building user trust and capacity by communicating state-of-the-art knowledge in regional climate models and being accessible as providers.

Trust is also built in the way climate projections and uncertainties are usefully and transparently presented. When engaging with KST users, climate projections are communicated by emphasising what is indeed known about the climate system and where models agree—the key messages—rather than focusing on knowledge gaps and the difficulties of creating future climate scenarios. This does not mean that information should be misleading; climate change information must be described correctly without concealing biases and uncertainties (e.g. Nissan et al. 2019). However, to improve usability, KST intends to present climate projection information as salient, credible, and legitimate (i.e. Jebeile 2024) as possible, without overwhelming users with uncertainty details. Whether the KST approach is beneficial for users still needs further and continued evaluation. Generally, there is a striking need to better incorporate evaluations into climate services processes, particularly regarding evaluations of the co-creation conducted and the benefits of using these services in decision-making and planning (e.g. Vaughan and Dessai 2014; Suckall and Soares 2022).

## Recommendations

**Fig. 3:**
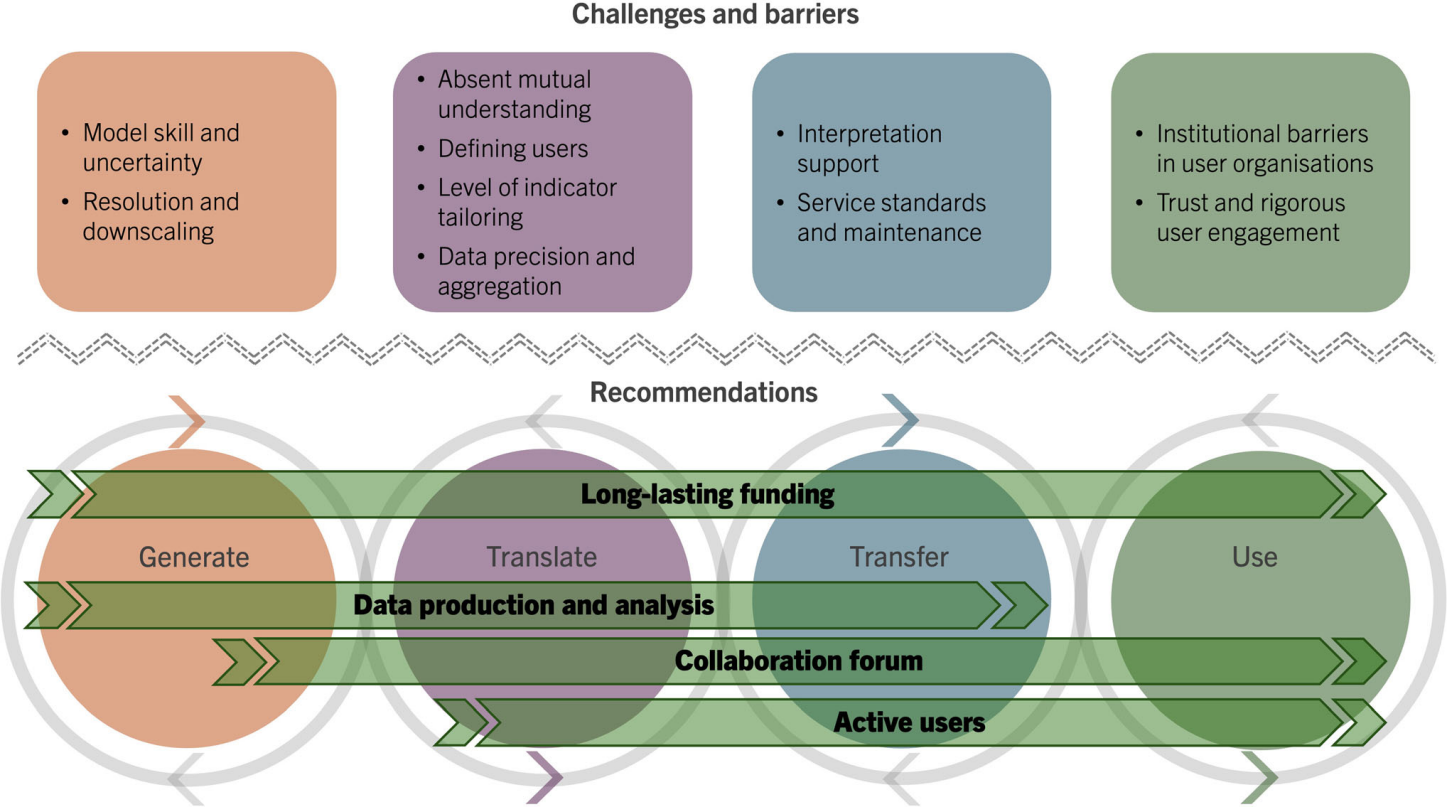
Four recommendations (arrows) to address climate service challenges and barriers (boxes) across the four pillars (circles)

The climate service community widely agrees that addressing the challenges and barriers related to usability requires the implementation of co-creation processes. However, we believe that the current conditions and organisations for this are insufficient. The following set (*i-iv*) of overarching recommendations (Fig. 3) constitutes a call for action beyond short-term studies and initiatives. If climate action in principle should be a natural part of all societal planning, steady and reliable climate information is essential. Pilot and shorter-term projects have been, and still are, important for demonstrating how co-creation of climate services can provide salient and legitimate climate information (Jebeile 2024). Nevertheless, for the benefit of society, research and policy must now also extend beyond that and focus on holistic approaches, including all four pillars and the operationalisation of climate services in society.

We recommend the following to national climate service institutes, policymakers, and other climate service stakeholders:

i. *Advancing Data Production and Analysis*—Maintain and advance the production, analysis, and evaluation of data to ensure high-quality results.

Addressing challenges associated with model uncertainties, ensemble spread, and model selection, as well as leveraging small ensembles or single high-resolution simulations while taking advantage of large RCM ensembles, requires continued and enhanced production and evaluation of climate data (Table 1). Increased computer power enables higher model resolution, which is known to improve model performance (Soares et al. 2022) but the computer power is still not enough to produce large model ensembles at a horizontal grid spacing of 5 km or less. Future efforts should focus on new ways of combining data of different resolutions to create large enough model ensembles for uncertainty estimates and statistical testing.

**Table 1:** How does the recommendation to “advance data production and analysis” (i) address climate service challenges and barriers

Pillar	Challenges and Barriers	Recommendation (i) specially address
Generate	Model skill and uncertainty	Support analysis of ensemble spread/uncertainties to facilitate model selections
	Resolution and downscaling	Balance the values of large RCM ensembles with small ensembles/single simulations of high-resolution models
Translate	Data precision and aggregation	More fully understand simulated climate data constraints and possibilities
Transfer	Interpretation support	High-quality data with understood uncertainties and limitations
	Service standards and maintenance	Ongoing processing and analysis of new simulations and updates

The production of new climate simulations and the rapid increase in data volume (as a result of increased resolution) make it critical to maintain and potentially even enhance the analysis and evaluation of climate models and their outputs to ensure data credibility. Possible shortcuts in production, such as leaving out technical quality control of the simulations, bias adjustment, or data validation, would negatively impact the data credibility and affect not only the *generate* pillar but the entire process, including all pillars.

 Our recommendation to advance data production and analysis also promotes better understanding of the constraints related to temporal and spatial climate data precision versus aggregations, ensuring information credibility while being relevant to users. Moreover, we envision that ensuring high-quality data through this recommendation will create a better basis for communicating the uncertainties and complexities of climate data, thereby supporting interpretation. While we consider the national climate service providers and policymakers as the primary target groups for this recommendation, other organisations and institutions involved in the climate data production and analysis efforts should continue their important contributions. This is preferably coordinated internationally, for example in CORDEX and CMIP, to make data consistent and comparable.

ii. *Establishing a Climate Service Collaboration Forum—*Institutionalising collaborations at national levels.
  We propose an institutionalised collaboration forum on climate services, including representatives from climate science, climate-resilient development research, business, and governmental agencies to address challenges related to all pillars (Table 2).

**Table 2:** How does the recommendation to “establish a collaboration forum” (ii) address climate service challenges and barriers

Pillar	Challenges and Barriers	Recommendation (ii) specially address
Generate	Resolution and downscaling	Involve users in prioritisation
Translate	Absent mutual understanding	Move beyond ad hoc and limited user engagement to foster learning through co-creation
		Build user capacity to comprehend climate information
		Build provider capacity to comprehend users’ needs and capacities
	Data precision and aggregation	Identify relevant and feasible data aggregations
	Defining users	Identify and prioritise among the potential users and their needs
	Level of indicator tailoring	Balance the details in indicators with the usability for the broader user group of National Climate Services
	Interpretation support	Facilitate communicating about complex data and uncertainties to be represented in a useful way
Transfer	Service standards and maintenance	Support the creation of common terminology and communication standards at the national level
Use	Institutional barriers in user organisations	Support the integration of climate action and climate information into general decision-making processes
	Trust and rigorous user engagement	Support enhancement of user engagement processes
  The idea of a collaboration forum can build upon previous and ongoing initiatives internationally, such as the Climate Service Partnership (CSP 2024) and the national consultation workshops suggested as part of establishing National Frameworks for Climate Services (WMO 2018). Another example to leverage is the Climate Services Summit suggested by the US Federal Framework and Action Plan for Climate Services, which is intended to bring together providers and users of climate information (Fast Track Action Committee on Climate Services 2023). However, while US Federal agencies have extensive stakeholder networks that they collaborate with in the context of climate services, similarly to SMHI in Sweden, it is not clear how the engagement should be conducted (ibid), highlighting the need for institutionalised collaborations.
  Co-creation takes time as it requires fostering of trust and mutual understanding, as well as defining the appropriate level of data tailoring for specific climate information user groups. Additionally, climate services should be dynamic and align with state-of-the-art climate science, including research on adaptation and mitigation. Therefore, the processes of translating and transferring climate data into meaningful data representations and feasible formats should be continuously and iteratively explored and evaluated in collaboration to ensure the information’s salience and legitimacy. While research repeatedly demonstrates the value of co-creation for usable climate service, we mean that this cannot solely rely on sporadic and ad hoc meetings by a national climate service provider or short-term research projects, instead it demands continuous and systematic approaches. To effectively support well-informed decisions across multiple levels of society, we argue that climate service collaborative engagements should be institutionally established at the national level. This means that the state must take responsibility for connecting different actors across the climate service pillars to ensure that the use of climate information is effective for climate actions, making this a recommendation both for national policymakers and national climate service providers.
iii. *Fostering Active Users*—Enhance the role of authorities, organisations, and businesses, to engage with climate information, supporting the development towards a climate-resilient society.
  Ultimately, the quality of climate data is of little importance if it is not used. It is crucial for users to recognise the benefits of understanding climate information and the value of climate projection data in their operating contexts. If not prioritised, we argue that climate information should at least be included as an important parameter in all society-related strategies, planning, and decision-making processes where weather or climate could have an impact. However, practical challenges in using and incorporating this information in various societal contexts often seem to be out of reach for the climate service community, particularly concerning barriers within users’ organisations, such as traditions, time constraints and capacity limitations (e.g. McNie 2013; Ernst et al. 2019; Wiréhn 2024).
  To address this and other challenges from the user perspective, we recommend assigning a clear overarching objective for climate service co-creation and use to a specific governmental agency (Table 3). This can facilitate ongoing engagement and help bridge the usability gap. We suggest appointing climate information knowledge brokers for specific user groups, ideally from different sectors, who can serve as user representatives in the *collaboration forum*. This knowledge broker approach could be inspired by the work of the Climate Knowledge Broker Group (Bauer and Smith 2015). However, we assert the need for such approach to be institutionalised at the national level.

**Table 3:** How does the recommendation to “foster active users” (iii) address climate service challenges and barriers

Pillar	Challenges and barriers	Recommendation (iii) specially address
Translate	Absent mutual understanding	Move beyond ad hoc and limited user engagement to foster co-creation and learning
		Build user capacity to comprehend climate information and what different data can offer
	Defining users	Identify users and their needs within sectors
Transfer	Service standards and maintenance	Support the communication of a common terminology at the national level
Use	Institutional barriers in user organisations	Support the integration of climate action and climate information into general decision-making processes
		Dedicated functions to engage with climate information at sectoral authorities, with allocated time and capacity
	Trust and rigorous user engagement	Making the value of engagement and climate information visible to the users and sectors
		Dedicated authority co-creation responsibility
  The key significance of this initiative is anticipated to be for the sectoral agencies that are informed by the brokers. These agencies can further facilitate the benefits of using climate information to the organisations and businesses in their sector, improve the climate information literacy, build trust in the climate information and services, and appoint specific roles within their sectors for functions who have the time, willingness, and capacity to integrate climate information into their planning and decision-making processes.
  Other benefits of assigning an overarching climate service co-creation and use objective to a specific governmental agency and sectoral knowledge brokers include feeding into the collaboration forum. The knowledge brokers can serve as sectoral representatives in the forum to co-define user groups, foster mutual understanding and learning, define relevant indicators and data aggregation levels, and create interpretation support among climate data providers and users.
iv *Prioritising Long-lasting Funding*—Ensure that the three former recommendations can be established.
  A long-term perspective is essential for providers, users, and other stakeholders of climate information to work iteratively in co-creation while addressing the outlined challenges and barriers through the adoption of the previous *i-iii* recommendations. The development, production, evaluation, and assessment of climate projections and services in co-creation are slow processes that demand commitment and continuity that goes beyond the typical time span of projects; the same applies to the management and maintenance of climate services. Given the challenges and barriers with climate service along with the notion that climate-resilient development should be underpinned by salient and credible climate information (e.g. IPCC 2022 TS.E; Malakar et al. 2024), we argue that sustained and long-lasting funding for climate services must be prioritised at both national and international levels to facilitate the establishment of the three previous recommendations (Table 4). While we do not have the expertise to say how this should be governed or funded, we envision a need to engage politicians in recognising the values of climate services, including all pillars, to secure funding that supports the gradual and sustained development of climate service infrastructures at national levels, in cooperation with international partners.

**Table 4:** How does the recommendation to “prioritise long-lasting funding” (iv) address climate service challenges and barriers

Pillar	Challenges and barriers	Recommendation (iv) specially address
Generate	Model skill and uncertainty	Support resource-intensive and time-consuming work
Translate	Data precision and aggregation	Ensure sustained co-production of tailored climate data
	Level of indicator tailoring	Ensure service management of climate indicator updates
Transfer	Service standards and maintenance	Support service maintenance under a constant inflow of data and updates
Use	Trust and rigorous user engagement	Support user engagement infrastructure
		Ensure continuity to build trust and demonstrate the benefits of engaging with climate information

## Conclusions

In this paper, we outline and discuss ten challenges and barriers that persist in the climate service community, despite the increased attention over the last decade to bridge the usability gap through co-creation. These challenges and barriers were identified from scientific literature, insights from stakeholder dialogues, and experiences from working with the Swedish climate change scenario service, KST.

We argue that addressing these constraints requires an increased prioritisation of climate services at multiple societal levels, speaking to *all* four pillars of climate services with a *long-term* perspective as the common denominator. While advocating for increased provider-user interaction and co-creation is essential, these processes need better facilitation, and moreover, co-creation alone is not sufficient. The development of high-quality data and collaborations on climate services must be integrated into national climate service processes. Given this need, it is crucial that these efforts are adequately valued, organised, and funded at the national level, as this is where the national climate services operate and what they should support. However, we recognise that the governance of the climate service policies involved warrants further research and careful evaluation before implementation.

We encourage national climate services to explore the identified challenges and consider how these impact the usability gap in their specific contexts. We envision that for climate service frontrunners, these recommendations can enhance existing initiatives, such as the governmental collaborations on climate adaptation in Sweden. For countries lacking climate service infrastructure, the recommendations can complement efforts related to their National Frameworks for Climate Services (WMO 2014, 2018), ensuring robust bottom-up co-creation from the outset. Ultimately, adopting these recommendations is our proposition of how to bridge the persistent usability gap through strengthened climate service processes and infrastructure, thereby enhancing the capabilities of all involved actors and serving the intended purpose of supporting decision-making for climate-resilient development.

## Data Availability

No new data were used for the research described in this article.

## Footnotes

1 “The transformation of climate-related data—together with other relevant information—into customised products such as projections, forecasts, information, trends, economic analysis, assessments (including technology assessment), counselling on best practices, development and evaluation of solutions and any other service in relation to climate that may be of use for the society at large. As such, these services include data, information and knowledge that support adaptation, mitigation and disaster risk management (DRM).” (European Commission et al. 2015, p. 10).

2 Definition on “Climate projections” in Hewitt et al. (2021).

3 “Klimatscenariotjänst” (KST) is the Swedish term for “Climate scenario service.”

## References

[CR1] Bauer, F., and J. Smith. 2015. *The climate knowledge brokers manifesto*. Vienna: Renewable Energy and Energy Efficiency Partnership (REEEP).

[CR2] Baulenas, E., D. Bojovic, D. Urquiza, M. Terrado, S. Pickard, N. González, and A.L. St Clair. 2023. User selection and engagement for climate services coproduction. *Weather, Climate, and Society* 15: 381–392. 10.1175/WCAS-D-22-0112.1.

[CR3] Berg, P., T. Bosshard, W. Yang, and K. Zimmermann. 2022. MIdASv0.2.1–multi-scale bias adjustment. *Geoscientific Model Development* 15: 6165–6180. 10.5194/gmd-15-6165-2022.

[CR4] Boon, E., S. Judith, R. Biesbroek, H. Goosen, and F. Ludwig. 2022. Successful climate services for adaptation: What we know, don’t know and need to know. *Climate Services* 27: 100314. 10.1016/j.cliser.2022.100314.

[CR5] Bremer, S., A. Wardekker, S. Dessai, S. Sobolowski, R. Slaattelid, and J. van der Sluijs. 2019. Toward a multi-faceted conception of co-production of climate services. *Climate Services* 13: 42–50. 10.1016/j.cliser.2019.01.003.

[CR6] Brunner, L., R. Lorenz, M. Zumwald, and R. Knutti. 2019. Quantifying uncertainty in European climate projections using combined performance-independence weighting. *Environmental Research Letters* 14: 124010. 10.1088/1748-9326/ab492f.

[CR7] Buontempo, C., H.M. Hanlon, M. Bruno Soares, I. Christel, J.-M. Soubeyroux, C. Viel, S. Calmanti, L. Bosi, et al. 2018. What have we learnt from EUPORIAS climate service prototypes? *Climate Services* 9: 21–32. 10.1016/j.cliser.2017.06.003.

[CR8] CSP. 2024. Climate services partnership. https://www.climate-services.org/index.php.en (Webpage) Accessed 2024-11-04.

[CR9] Dilling, L., and M.C. Lemos. 2011. Creating usable science: Opportunities and constraints for climate knowledge use and their implications for science policy. *Global Environmental Change* 21: 680–689. 10.1016/j.gloenvcha.2010.11.006.

[CR10] DMI. 2024. Klimatatlas. *Nationalt Center for Klimaforskning, Danmarks Meteorologiske Institut*. (Webpage) https://www.dmi.dk/klimaatlas/Accessed 2024-11-06.

[CR11] Donnelly, C., K. Ernst, and B. Arheimer. 2018. A comparison of hydrological climate services at different scales by users and scientists. *Climate Services* 11: 24–35. 10.1016/j.cliser.2018.06.002.

[CR12] DWD. 2024. Climate Predictions for Germany. *ESGF Node at Deutscher Wetterdienst*. (Webpage) https://esgf.dwd.de/projects/climatepredictionsde/Accessed 2024-11-06.

[CR13] Ernst, K.M., Å.G. Swartling, K. André, B.L. Preston, and R.J.T. Klein. 2019. Identifying climate service production constraints to adaptation decision-making in Sweden. *Environmental Science and Policy* 93: 83–91. 10.1016/j.envsci.2018.11.023.

[CR14] European Commission, Directorate-General for Research and Innovation, R. Street, M. Parry, J. Scott, D. Jacob, and T. Runge. 2015. *A European research and innovation roadmap for climate services*. Publications Office of the European Union. 10.2777/702151.

[CR15] Fast Track Action Committee on Climate Services. 2023. *A federal framework and action plan for climate services*. Washington: The Office of Science and Technology Policy.

[CR200] Filho, W.L. 2020. Introducing climate services and their applications. In *Handbook of climte services*, eds. W. Leal Filho, D. Jacob, 3–9.

[CR16] Findlater, K., S. Webber, M. Kandlikar, and S. Donner. 2021. Climate services promise better decisions but mainly focus on better data. *Nature Climate Change* 11: 731–737. 10.1038/s41558-021-01125-3.

[CR17] Grossi, A., and T. Dinku. 2022. Enhancing national climate services: How systems thinking can accelerate locally led adaptation. *One Earth* 5: 74–83. 10.1016/j.oneear.2021.12.007.

[CR18] Guido, Z., C. Knudson, D. Campbell, and J. Tomlinson. 2020. Climate information services for adaptation: What does it mean to know the context? *Climate and Development* 12: 395–407. 10.1080/17565529.2019.1630352.

[CR19] Hansen, J.W., C. Vaughan, D.M. Kagabo, T. Dinku, E.R. Carr, J. Körner, and R.B. Zougmoré. 2019. Climate services can support African farmers’ context-specific adaptation needs at scale. *Frontiers in Sustainable Food Systems* 3: 21. 10.3389/fsufs.2019.00021.

[CR20] Hawkins, E., and R. Sutton. 2009. The potential to narrow uncertainty in regional climate predictions. *Bulletin of the American Meteorological Society* 90: 1095–1108. 10.1175/2009BAMS2607.1.

[CR21] Hewitt, C.D., F. Guglielmo, S. Joussaume, J. Bessembinder, I. Christel, F.J. Doblas-Reyes, V. Djurdjevic, N. Garrett, et al. 2021. Recommendations for future research priorities for climate modeling and climate services. *Bulletin of the American Meteorological Society* 102: E578–E588. 10.1175/BAMS-D-20-0103.1.

[CR22] IPCC. 2021. *Climate change 2021: The physical science basis. Contribution of working group I to the sixth assessment report of the intergovernmental panel on climate change*. Edited by V Masson-Delmotte, P Zhai, A Piani, S.L Connors, C. Péan, S. Berger, N. Caud, L. Goldfarb, et al. Cambridge: Cambride University Press.

[CR23] IPCC. 2022. *Climate change 2022–impacts, adaptation and vulnerability: Working group II contribution to the sixth assessment report of the intergovernmental panel on climate change*. Edited by H.-O H.-O. Pörtner, D.C. Roberts, M. Tignor, E.S. Poloczanska, K Mintenbeck, A Alegría, S Craig, S Landsdorf, et al. 1st ed. Cambridge: Cambridge University Press. 10.1017/9781009325844.

[CR24] IRI. 2024. Adapting agriculture to climate today, for tomorrow. *International Research Institute for Climate and Society*. (Webpage) https://iri.columbia.edu/actoday/ Accessed 2024-05-27.

[CR25] Jacob, D., C. Teichmann, S. Sobolowski, E. Katragkou, I. Anders, M. Belda, R. Benestad, F. Boberg, et al. 2020. Regional climate downscaling over Europe: Perspectives from the EURO-CORDEX community. *Regional Environmental Change* 20: 51. 10.1007/s10113-020-01606-9.

[CR26] Jacobs, K.L., and R.B. Street. 2020. The next generation of climate services. *Climate Services* 20: 100199. 10.1016/j.cliser.2020.100199.

[CR27] Jebeile, J. 2024. From regional climate models to usable information. *Climatic Change* 177: 53. 10.1007/s10584-024-03693-7. 38434209 10.1007/s10584-024-03693-7 PMC10904437

[CR28] Kalsnes, B., A. Oen, R. Frauenfelder, I. Heggelund, M. Vasbotten, B. Vollstedt, J. Koerth, N. Vafeidis, et al. 2023. Stakeholder evaluation of the co-production process of climate services. Experiences from two case studies in Larvik (Norway) and Flensburg (Germany). *Climate Services* 32: 100409. 10.1016/j.cliser.2023.100409.

[CR29] Kirchhoff, C.J., M.C. Lemos, and S. Dessai. 2013. Actionable knowledge for environmental decision making: Broadening the usability of climate science. *Annual Review of Environment and Resources* 38: 393–414. 10.1146/annurev-environ-022112-112828.

[CR201] Kjellström, E., L. Bärring, G. Nikulin, C. Nilsson, G. Persson, and G. Strandberg. 2016. Production and use of regional climate model projections – A Swedish perspective on building climate services. *Climate Services* 2–3: 15–29. 10.1016/j.cliser.2016.06.004. 10.1016/j.cliser.2016.06.004 PMC5351818 28345063

[CR30] Kundzewicz, Z.W., E.J. Førland, and M. Piniewski. 2017. Challenges for developing national climate services–Poland and Norway. *Climate Services* 8: 17–25. 10.1016/j.cliser.2017.10.004.

[CR31] Lemos, M.C., C.J. Kirchhoff, and V. Ramprasad. 2012. Narrowing the climate information usability gap. *Nature Climate Change* 2: 789–794. 10.1038/nclimate1614.

[CR202] Mahon, R., C. Greene, S.A. Cox, Z. Guido, A.K. Gerlak, J.A. Petrie, A. Trotman, D. Liverman, C.J. Van Meerbeeck, W. Scott, and D. Farrell. 2019. Fit for purpose? transforming national meteorological and hydrological services into national climate service centers. *Climate Services* 13: 14–23. 10.1016/j.cliser.2019.01.002.

[CR32] Malakar, Y., S. Snow, A. Fleming, S. Fielke, E. Jakku, C. Tozer, and R. Darbyshire. 2024. Multi-decadal climate services help farmers assess and manage future risks. *Nature Climate Change*. 10.1038/s41558-024-02021-2.

[CR33] Máñez Costa, M., A. M. P. Oen, T.-S. Neset, L. Celliers, M. Suhari, J.-T. Huang-Lachmann, R. Pimentel, B. Blair, et al. 2021. Co-production of climate services. CSPR Report Series. Centre for climate Science and Policy Research. 10.3384/9789179291990.

[CR34] Mauser, W., G. Klepper, M. Rice, B.S. Schmalzbauer, H. Hackmann, R. Leemans, and H. Moore. 2013. Transdisciplinary global change research: the co-creation of knowledge for sustainability. *Current Opinion in Environmental Sustainability* 5: 420–431. 10.1016/j.cosust.2013.07.001.

[CR35] McNie, E.C. 2013. Delivering climate services: Organizational strategies and approaches for producing useful climate-science information. *Weather, Climate, and Society* 5: 14–26. 10.1175/WCAS-D-11-00034.1.

[CR36] NCCIS. 2024. National climate change information system. *Department of Forestry, Fisheries and the Environment, Republic of South Africa*. (Webpage) https://gisportal.saeon.ac.za/portal/apps/webappviewer/index.html?id=2d572dcf9c5f47c484540f8c934e03f4 Accessed 2024-11-06.

[CR37] Nissan, H., L. Goddard, E.C. de Perez, J. Furlow, W. Baethgen, M.C. Thomson, and S.J. Mason. 2019. On the use and misuse of climate change projections in international development. *Wiley Interdisciplinary Reviews: Climate Change* 10: 1–16. 10.1002/wcc.579.

[CR38] SMHI. 2024. Advanced climate change scenario service. *The Swedish Meteorological and Hydrological Institute Research Department*. (Webpage) https://www.smhi.se/en/climate/future-climate/advanced-climate-change-scenario-service/met Accessed 2024-05-20.

[CR39] Smid, M., and A.C. Costa. 2018. Climate projections and downscaling techniques: A discussion for impact studies in urban systems. *International Journal of Urban Sciences* 22: 277–307. 10.1080/12265934.2017.1409132.

[CR40] Soares, P.M.M., J.A.M. Careto, R.M. Cardoso, K. Goergen, E. Katragkou, S. Sobolowski, E. Coppola, N. Ban, et al. 2022. The added value of km-scale simulations to describe temperature over complex orography: The CORDEX FPS-convection multi-model ensemble runs over the Alps. *Climate Dynamics*. 10.1007/s00382-022-06593-7.

[CR41] Strandberg, G., P. Blomqvist, N. Fransson, L. Göransson, J. Hansson, S. Hellsten, E. Kjellström, C. Lin, et al. 2024. Bespoke climate indicators for the Swedish energy sector−a stakeholder focused approach. *Climate Services* 34: 100486. 10.1016/j.cliser.2024.100486.

[CR42] Suckall, N., and M.B. Soares. 2022. Evaluating the benefits of weather and climate services in South Asia: A systematic review. *Regional Environmental Change* 22: 104. 10.1007/s10113-022-01947-7.

[CR43] Tanimu, B., A.-A. Danladi Bello, S.A. Abdullahi, M.A. Ajibike, M.K. Idlan Bin Muhammad, and S. Shahid. 2024. Enhancing reliability in climate projections: A novel approach for selecting global climate models. *Physics and Chemistry of the Earth* 134: 103598. 10.1016/j.pce.2024.103598.

[CR44] Tebaldi, C., and R. Knutti. 2007. The use of the multi-model ensemble in probabilistic climate projections. *Philosophical Transactions of the Royal Society a: Mathematical, Physical and Engineering Sciences* 365: 2053–2075. 10.1098/rsta.2007.2076. 10.1098/rsta.2007.2076 17569654

[CR45] Vaughan, C., and S. Dessai. 2014. Climate services for society: Origins, institutional arrangements, and design elements for an evaluation framework. *Wiley Interdisciplinary Reviews: Climate Change* 5: 587–603. 10.1002/wcc.290. 25798197 10.1002/wcc.290 PMC4362079

[CR46] Vautard, R., N. Kadygrov, C. Iles, F. Boberg, E. Buonomo, K. Bülow, E. Coppola, L. Corre, et al. 2021. Evaluation of the large EURO-CORDEX regional climate model ensemble. *Journal of Geophysical Research: Atmospheres* 126: e2019JD032344. 10.1029/2019JD032344.

[CR47] Vincent, K., M. Daly, C. Scannell, and B. Leathes. 2018. What can climate services learn from theory and practice of co-production? *Climate Services* 12: 48–58. 10.1016/j.cliser.2018.11.001.

[CR48] Wiréhn, L. 2024. From relevant to usable: Swedish agricultural extension officers’ perspectives on climate change projections. *Climate Services* 33: 100441. 10.1016/j.cliser.2023.100441.

[CR49] WMO. 2014. *Implementation plan of the global framework for climate services*. *Wmo*. vol. 2. WMO.

[CR50] WMO. 2018. *Step-by-step guidelines for establishing a national framework for climate services*. Vol. 2018. 1206. Geneva, Switzerland: World Meteorological Organization (WMO).

